# The toxic effects of subjective wellbeing and potential tonics

**DOI:** 10.1016/j.socscimed.2020.113098

**Published:** 2021-11

**Authors:** Sarah Atkinson

**Affiliations:** Durham University, Department of Geography and Institute for Medical Humanities, Lower Mountjoy, Durham, DH1 3LE, United Kingdom

**Keywords:** Wellbeing, Collective, Inequality, Sustainability, Generational, Relational

## Abstract

The paper offers a provocation to the geographies of health in relation to one of our governing concepts, that of wellbeing. The paper brings together government survey data from the United Kingdom with other published research into a critical argument that the dominant ways of conceptualising and practising subjective wellbeing have become toxic and harmful to wellbeing outcomes. The paper argues that a ‘hyper-individualised and thwarted self’ and ‘supermarket model’ of social resources for individual wellbeing underpins the contemporary dominant understanding of subjective wellbeing. This approach neglects wider spatial and temporal considerations such as inequality, inter-generationality and sustainability, and the rise of wellbeing as a technology of soft capitalism. The paper discusses the potential for relational approaches from the social sciences to provide a more ‘wholesome tonic’ to current understandings of subjective wellbeing that might rehabilitate its capability to do helpful rather than harmful work and argues for an ethical obligation to sustain critical engagement.

## Highlights

• The dominant understanding of subjective wellbeing creates negative consequences.
• Direct effects reduce wellbeing; indirect effects neglect importance issues.
• This dominant approach reflects and supports a political economy of soft capitalism.
• Centring the relational, social and interdependent fails to gain policy traction.
• Struggling to reframe subjective wellbeing is an ethical imperative.

## 1 Introduction

This paper offers a provocation to the Geographies of Health in relation to one of our most cherished concepts, that of wellbeing, and specifically the sense of our own wellbeing, referred to here as subjective wellbeing. The paper combines government survey data on subjective wellbeing from the United Kingdom with other published conceptual and empirical work into a critical essay that builds a two-stage argument. First, the contemporary dominant understanding of subjective wellbeing is very narrow and overly centred on the individual agent. Secondly, this dominant way of thinking results in practices that undermine the very thing we seek to enable in that the implications for popular imagining of our selves and our lives render subjective wellbeing a harmful and toxic concept. As such, my provocation to colleagues in the geographies of health and the wider family of social scientists and health researchers is to ask whether we should jettison thinking and working with the concept of subjective wellbeing altogether or whether there may still be room for its rehabilitation. The paper offers as potential ‘wholesome tonics’ those approaches across the social sciences that engage relationality (Gergen, 2009; White, 2017), including geographical concerns with settings, scales and time.

Measuring aspects of wellbeing, albeit under a variety of names and indicators, is not new in policy, philosophy or social and spatial science (see Conradson, 2012 for a history of wellbeing in Geography). What is new is the rapid growth of interest in capturing subjective wellbeing, that is, those aspects of wellbeing that overlap with concepts such as happiness and purpose in life, and in using measurable indicators as part of assessing social progress (see Stiglitz et al., 2009).

## 2 British trends in subjective wellbeing

In the United Kingdom, the Office for National Statistics (ONS) has monitored subjective wellbeing since 2011 through four indicators: three measuring positive wellbeing as feeling satisfied, worthwhile and happy, and one measuring negative wellbeing as anxiety (ONS, 2019a). The approach is efficient in recognising and trying to capture different nuanced understandings of wellbeing and in acknowledging positive and negative affect as separate dimensions rather than poles of a single spectrum (Huppert and Whittington, 2003). Since 2011, the data describe a trend of modest steady improvements, a plateau in 2016–17 (ONS, 2017a), and some further improvement in 2018–19 (ONS, 2019a). The ONS’ own speculative suggestions for the wellbeing trends relate to economic recovery from the 2008 recession, including increased wage levels and a thirty-year low in unemployment (ONS, 2017a, 2018).

The figures for the third quarter of 2019 (July to September), however, showed a downward turn for the first time since 2011 for life satisfaction and feeling worthwhile, although they were stable over the fourth quarter (October–December) (ONS, 2020a; 2020b). The ONS relate this to perceptions of potential economic uncertainty and job insecurity despite objective measures of employment remaining high (ONS, 2020b). The second half of 2019 was a notably turbulent and uncertain time in the political process of the UK's departure from the European Union, characterised by repeated crises between government and parliament. Nonetheless, the absolute levels of subjective wellbeing remain substantially above the starting levels in 2011 (ONS, 2020a) and describe a largely positive picture of population subjective wellbeing in the UK. This has changed in 2020 with the unprecedented disruption of the COVID-19 pandemic and all four measures show clear declines (ONS, 2020c).

The ONS’ interpretations for wellbeing change through the activities and perceptions of the wider economy are interesting on several points. ONS (ONS, 2020a) cite a paper by Knabe and Rätzel (2008) on the importance of the fear of insecure future employment as a determinant of life satisfaction, although, surprisingly, not anxiety. This argument places subjective wellbeing firmly within the domains of both national and global economic processes and government policy rather than local individual and community management. This mode of proposed possible explanation, however, reverts to positioning the economy as the primary driver of wellbeing which, regardless of whether actual performance or perceived confidence is referenced, effectively undermines the argument for measuring subjective wellbeing in the first place. The influence of perceived confidence in a future economic performance and associated job security foregrounds questions of how subjective wellbeing relates to time, something rarely discussed explicitly (Atkinson et al., 2019). Finally, falling back onto economic explanations also treats as unproblematic how national statistics capture collective subjective wellbeing, the being well together of a nation. This is problematic as demonstrated by how wellbeing measures largely miss the political distress evident in the UK from 2016 and expressed through contested claims of heritage and belonging, social affiliations and exclusions, that is, whose wellbeing matters (Atkinson et al., 2019).

National trends in subjective wellbeing before COVID-19 thus describe a largely positive picture, but there are plenty of other data suggesting the opposite; that social life in the United Kingdom is not proceeding at all well (Easton, 2020; Hansard Society, 2019; Ipsos-MORI, 2019). First, there is considerable fall-out from the 2016 referendum on whether to leave the European Union (Powdthavee et al., 2019). Possibly the only point of agreement regarding the referendum is its disclosure of deep-seated fractures in social priorities and values within the country along lines of class, education, generation, geography, and history (BBC, 2016; Electoral The Electoral Commission, 2019; Statistica, 2019a, 2019b; YouGov, 2016). In the aftermath of the referendum, these fractures have manifested themselves through political turmoil and social antagonisms, including an increase in racialised and gendered hatred and hate-crimes (Home Office, UK, 2019). Secondly, the political landscape is characterised by a set of parallel narratives of crisis including those related to the environment, chronic shortage of affordable housing (ONS, 2019b), and an underfunded and understaffed system of health and social care provision (RCN, 2018; Triggle, 2018). The COVID-19 response has affirmed the last of these (Gregory et al., 2020) while its risk and impact has mirrored existing health differentiation of age, socio-economic status and ethnicity (Booth and Barr, 2020) and existing fractures of xenophobic and racist abuse (Mercer, 2020). Thirdly, there has been a rapid rise in numbers of people illegally trafficked into the country (HM Government, 2018; Tourangeau et al., 2014) who have negligible wellbeing and fall below the radar of wellbeing assessment. Fourthly, young people show a significant increase in mental ill-health (Booth, 2019; Bulman, 2017; Departments of Health and Social Care for Education, 2018; NHS Digital, 2018; Pitchforth et al., 2019; Prince's Trust, 2019), particularly young women, although this partly reflects their greater willingness to seek assistance (NHS Digital, 2018). Finally, the 2019 Ipsos-MORI poll of mood (2019) and the annual Edelman Trust Barometer (2020) both report particularly low levels in the UK for optimism and confidence and for trust respectively. The CEO of the Europe, Middle East and Africa Region for Edelman, Ed Williams, commented following the 2020 report:

“A toxicity has overtaken the tone of the UK's internal conversation. Politicians of every party, including the Prime Minister, have taken to attacking the BBC, which other countries regard as a beacon of impartiality, for its bias. Other parts of the UK media openly attack the judiciary in a nation where the separation of powers has been a given for four centuries. And everyone, from MPs to schoolchildren, has taken to attacking each other on social media.” [Williams, 2020]

These data present an important contradiction between the national trends in subjective wellbeing and national figures for other manifestations of how life is going in the United Kingdom, including aspects encompassed by wellbeing such as mental health and trust, positivity and future prospects. As such, the first claim in the argument of this paper, that how we assess subjective wellbeing is toxic, is that there seems to be a serious risk that the measurements fail to detect with sufficient sensitivity or speed the very thing they aim to describe. It is difficult to see how the country, given the pre-COVID-19 state of division and turmoil, can have generated a national assessment of subjective wellbeing characterised predominantly by an improving trend and a good absolute level. There is a need, therefore, to reflect on what work it is that we do in assessing wellbeing and on what it is about how we do this that results in such a surprisingly complacent description of societal wellbeing.

### 2.1 The hyper-individualised and thwarted self of subjective wellbeing

The argument made for investing in measuring subjective wellbeing is that the most important goal for all people is not material but how we feel about how our lives are going in terms of whether we are, on balance, happy, satisfied and feeling worthwhile. The ever-growing body of research on defining the concepts, the indicators and the determinants of subjective wellbeing serves to gainsay the deceptive simplicity of this apparent self-evident truism. Historical (Conradson, 2012; Sointu, 2005), cross-cultural (White and Blackmore, 2016) and cross-disciplinary reflections (White and Jha, 2018) all attest to the different possibilities for conceptualising, assessing or supporting wellbeing, a variation that has largely become hidden from current practices.

The contemporary dominant approach to individual subjective wellbeing, and how this translates into a collective assessment, builds on a particular understanding of the self as a largely independent, autonomous and intentional individual, including in our social relations. This characterisation of the self is documented in political theory as emergent with modernity and capitalism and consolidated within regimes of neoliberalism (Miller and Rose, 2008; Sointu, 2005). The workforce, in what has been termed reflexive or ‘liquid’ modernity (Bauman, 2005), is no longer seen as occupational groups of labourers but as individuated human capital (Berg et al., 2016). As such, each of us is responsible for reflexivity and investment into the self to manage risks and uncertainties, albeit within a pre-defined and narrow set of goals (Smith and Vonthethoff, 2017; Thrift, 1997). These shifts in organisational management, often captured under the moniker of ‘soft capitalism’ (Costea et al., 2008; McCormack and Salmenniemi, 2016; Thrift, 1997), mobilise wellbeing as part of performing a competent self. The culture of constant improvement and optimisation, however, renders this self as always and necessarily beyond reach and work to improve the self effectively pursues a potential for wellbeing rather than securing wellbeing (Bauman, 2005). Paradoxically, while this self is incomplete and thwarted in its authentic expression, it is simultaneously the source of its own potential fulfilment through appropriate self-work (Mäkinen, 2014). The thwarted self of subjective wellbeing underpins a symbiotic relationship between soft capitalism and academic champions that draws on economic utility (Layard, 2005) and positive psychology (Seligman, 2011). Positive psychology, in particular, advances an argument that optimism or positive thinking is associated with a range of other desirable outcomes, including the social trappings of success, and that this desirable, optimistic outlook can be learned.

The growth of interest in the internal processes of mind, emotion and pre-cognition, and particularly in neuroscience, has deepened this way of thinking about our selves further in what Whitehead and colleagues term ‘neuroliberalism’ (Whitehead et al., 2019). Accounts of social ills as grounded in individual failings are furthered through new research linking brain structure, anti-social behaviours and poor wellbeing (Pykett, 2015; Rose and Abi-Rached, 2014; Whitehead et al., 2019). This effectively reconfigures both poor individual wellbeing and inequalities in collective wellbeing as personal, rather than social or political. This intensification of attention to individual interiority appears to face a counter-movement in the growth of interest in the post-human condition including, amongst other things, to new and mobile technologies (Andrews, 2018). Academics often see these new relations, hybrid selves and spatial reconfigurations as celebrating a decentred self. There is, however, an alternative perspective on the rapid market growth of self-monitoring mobile technologies. These technologies allow us increasingly to track our experiences moment-by-moment, step-by-step, mood-by-mood, and, in doing so, we might consider these technologies as effectively hyper-centring and reconstructing ourselves as both hyper-individualised, thwarted selves and quantified selves (Lupton, 2017). In research, such technologies enable biosensing physiological responses associated with emotions (see Aspinall et al., 2015), analysis of social media posts (see Zeile et al., 2015), prompts for the immediate recording of experience (see Hurlburt, 2017) or correlations of geolocation data with physiological measures (Tost et al., 2019). In our everyday lives, fit-bits and apps, such as mindspace, enable constant monitoring of fitness regimes, physiological responses, daily activities and personal mood, which their users value for self-improvement, exerting control and achieving goals (Lupton and Smith, 2018). The self-tracking technologies both enable and respond to the imperative to direct attention onto our own selves as the source of our wellbeing and our interior selves as the place for intervention within a wider political economy of soft capitalism and liquid modernity.

The relocation of the site for intervention in subjective wellbeing onto the interior self displays two further features that increasingly dominate our assumptions in conceptualising subjective wellbeing. The new technologies focus on capturing the micro-changes and micro-temporalities of the inner self and thereby privilege the moment, the here and now, as the most authentic account of experience, emotion, cognition and our associated wellbeing; measurement becomes about how to capture the immediacy of that moment of experience. At the same time, techniques to improve wellbeing often effectively depict our engagements with others as interfering, distracting and distorting the expression of our true selves (Whippman, 2016). This is perhaps most evident in the underpinnings of a range of technologies of ‘new spirituality’ (Philo et al., 2015), such as mindfulness, meditation or yoga, which explicitly practice techniques to shut out externalities in order to rediscover and recover an internal and authentic self (Whippman, 2016).

These various elements thus build and advance an understanding of the self as hyper-individualised and thwarted, as always and of necessity unfulfilled and of self-care for wellbeing as an unending pursuit of recovery, nurture and optimisation of a potential but authentic self. The policy expression of this shift to responsibility for the care of our own and close others' wellbeing has several critical responses. The most important of these is that relocating responsibility for subjective wellbeing onto individuals themselves detracts political attention and intervention away from the deep-rooted structural inequalities and social determinants of wellbeing. Moreover, in workplace settings, wellbeing conflates easily with career success and satisfaction, which, in turn, conflate with a given, or predefined, range of attitudes and desires. These conflations effectively reconstruct wellbeing from fitness to fitness for purpose (Dale and Burrell, 2014), combine different forms of individual potential with labour market values (Mäkinen, 2016) and blur an entitlement to wellbeing with a duty to wellbeing (Costea et al., 2005). Such conflation prompts those already disadvantaged to invest limited resources into personal development as the promised solution for a lack of career success (Ehrenreich, 2009), or justifies the coercion of unemployed recipients of welfare support to attend attitudinal retraining to access their benefit entitlements (Friedli and Stearn, 2015). Beyond the workplace, the hyper-individualised and thwarted self in need of self-care supports the rapid growth of a commercialised industry to assist the quest (Davies, 2015), iconically captured by L'Oreal's marketing catch-line, ‘because you're worth it’. Finally, the conflations of wellbeing within an economic and social context of inequality, precarity and competition drive Berlant's (2011) concept of ‘cruel optimism’: the promise and potential for being well in a different present combined with the process of being worn down by the realities of effort (Jokinen, 2016; McCormack and Salmenniemi, 2016). As Braithwaite (2004: 13) comments, ‘to hope in a world that is not responsive is a tall order.’

### 2.2 A supermarket approach to social relations

Subjective wellbeing, premised on a hyper-individualised and thwarted self, informs how we assess collective wellbeing, which usually aggregates individual assessments into population level figures. This practice carries three important implications. First, this approach only works for defined territorial areas or specific social groups. The reality of lives lived across a number of different settings, such as those of residence, work, leisure, friendship or online networks, is overlooked as there is no bounded group of people across which easily to aggregate individual scores. Secondly, it exposes an inability and relative absence of effort to recognise and capture the more-than-individual quality that we take as part of collective wellbeing. The notion of collectivity, territorial affiliation or community conveys a sense of a social unit that is more than the sum of its parts through networks of affiliation. Lastly, the dual facets of individualised wellbeing and a politics of individual responsibility combine to deliver a shift downwards of responsibility for collective wellbeing to local governance and civic organisations in relation to local issues and strategies (Scott, 2015). These processes, and their implications for policy, thus relocate where we think politically about wellbeing and neglect wellbeing as embedded in wider structures of politics and inequality and as shaped by factors operating across a range of spatial and temporal scales (Atkinson et al., 2019).

Social relations, regardless of how they are conceptualised, labelled or mobilised, promise a way into capturing that elusive relationality that makes collective wellbeing about more than an aggregate of the constituent individuals. In reality, however, the practice of collecting and aggregating individual reports of social networks, activities and values reduces these social affiliations to determinants of individual wellbeing. The dominant emphasis on the hyper-individualised, thwarted self extends, therefore, into undermining the ways that we are able to think about social, collective and community in relation to subjective wellbeing. What should be a central and nuanced concept of social relations for understanding the ways in which we succeed or fail in being well together becomes little more than a set of resources for individual wellbeing. This effectively constructs a supermarket model of collective wellbeing in which any notion of community, sociality or relationality reduces to something similar to a pick-n-mix counter and in which social or civic outcomes are secondary to the sovereignty of individual wellbeing. To stress the point here, having friends, joining clubs, feeling safe or a sense of belonging are important because these are beneficial to our individual wellbeing; what is clearly missing is the value of social association in and for itself.

The ONS, who have a strong interest in social capital and who do endeavour to identify collective measures of people's engagements and connections, illustrate how difficult it is to move beyond aggregated individual level scores. They, however, reveal a ‘supermarket model’ of social affiliation underpinning their approach,

‘Our personal relationships can be a source of enjoyment and happiness in our lives and provide a sense of comfort and stability. Research shows that health personal relationships can be a protective factor against stress and other health issues.’ (ONS, 2017c: 6).

The ONS suite of collective level indicators (see Table 1) offer three kinds of measure: aggregated individual scores on personal resources; aggregated individual scores on perceptions of the social context; and area statistics on local social engagement, specifically voting turn-out, which is not really a measure of subjective wellbeing. The second category may be the best that conventional indicator-led assessments can offer for collective wellbeing.

**Table 1:** ONS indicators for social capital.

Indicators	Latest Data National %
*Personal Relationships*	
1. At least one close friend	97
2. Meet socially with friends, relatives of work colleagues at least once a week	61
3. Feelings of loneliness often/always^a^	4
4. Used the internet for social networking in the last three months	63
5. Regularly stop and talk with people in the neighbourhood	68
*Social Support Networks*	
6. Have a spouse or partner, family member or friend to rely on a lot if they have a serious problem	84
7. Give special help to at least one sick, disabled or elderly person living or not living with them	20
8. Parents who regularly receive or give practical or financial help from/to a child aged 16 or over not living with them	Receive 38 Give 58
9. Borrow things and exchange favours with their neighbours	42
*Civic Engagements*	
10. Volunteered more than once in the last twelve months	19
11. Members of organisations, whether political, voluntary, professional or recreational	53
12. Involved in at least one social action project in the local area in the previous 12 months^a^	18
13. Definitely agree or tend to agree that they can influence decisions affecting their local area^a^	36
14. Voter turn-out in the UK General Elections	66
15. Involved in at least one political action in the previous 12 months	34
16. Very or quite interested in politics	56
*Trust*	
17. Have trust in national Government	35
18. Say that most people can be trusted	35
19. Say that most people in their neighbourhood can be trusted	70
20. Definitely agree or tend to agree that their local area is a poace where people from different backgrounds get on well together	89
21. Felt fairly/very safe walking along after dark^b^ (men/women)	88/62
22. Agree or strongly agree that people around where they live are willing to help their neighbours	74
23. Agree or strongly agree that they feel they belong to their neighbourhood	69

Adapted from ONS (2017a, 2017b, 2017c) Social capital in the UK. London: ONS, May 2017.

a England only.

b England and Wales only.

### 2.3 Toxic wellbeing

The proposition that the dominant mode of mobilising subjective wellbeing may be more harmful than helpful predicts toxic impacts at individual, collective, organisational and national scales. Teasing out the complexities of these relationships is only rarely and partially undertaken. Nonetheless, three types of study are producing evidence on the toxic effects from privileging this particular form of subjective wellbeing: direct measurement; specific case studies; neglected aspects of wellbeing.

Direct evidence that how we construct wellbeing may harm wellbeing is emerging from experimental psychological research, which has demonstrated an association between valuing wellbeing and negative wellbeing outcomes. The social pressure not to experience or express negative emotions shows association with those very things and can act as a predictor of depressive symptoms (Dejonckheere et al., 2017; Ford et al., 2014). While much of this work is correlational rather than explanatory, experimental research indicates that pursuing wellbeing influences negative outcomes rather than the other way around (Ford and Mauss, 2014). These negative effects are more marked in highly individualistic societies (Ford et al., 2015) which pursue hedonic pleasure over the social harmony pursued by collectivist cultures (Gruber et al., 2011). The blanket pursuit of harmonious collaboration, however, including when confrontation might be more appropriate, also showed lower wellbeing outcomes (Tamir and Ford, 2012). Explanations for these paradoxical effects of the valuing of happiness relate to an expectation-reality gap, resonating with Berlant's ‘cruel optimism’, and to inaccurate judgements. Lowered wellbeing is particularly evident when valuing wellbeing both raises expectations and prompts monitoring, thereby foregrounding any discrepancies. People do not always judge accurately what will increase subjective wellbeing and may not always make good choices (Ford and Mauss, 2014). This observation underpins the new enthusiasm for behavioural economics and ‘nudge’ approaches in policy (Dolan, 2014; Thaler and Sunstein, 2008) and a shift from a laissez-faire approach of individual preferences expressed through a free market to libertarian paternalism in which interventions through small adjustments encourage individuals to make pre-defined, politically desirable choices (Whitehead et al., 2019).

Case studies of organisations can explore the connections between changing forms of capitalism, managerial practices, employee subjectivities, and the place, role and outcomes of subjective wellbeing within these. A critical analysis of this kind across academic institutions in five country settings in Northern Europe reports common management and goals of soft capitalism and soft governance across the different national settings, despite differentiated historical and political trajectories. The experiences of the academic community are impacted by increased demands and lowered control over outputs, the application of auditing and accountancy systems, the blurring of work with other domains of life and the replacement of a work environment characterised by collegiality by one of greater competitiveness. In the context of academic work, the production of knowledge shifts from use value to exchange value, with the loss of its potential for satisfaction for its own sake. The researchers argue that the effects of these various differentiating ‘political technologies’ of soft capitalism underlie the increased anxiety both in academia and in other sectors (Berg et al., 2016).

Berg and colleagues intentionally seek commonalities across different settings, aware that the expression of the wider cultures of political economy will always be geographically uneven and temporally contingent. A third expression of the toxicity of how we understand subjective wellbeing is through the neglect of exactly these contextual aspects of geographical and temporal unevenness. The neglect of direct attention to inequality as a measure of subjective wellbeing is particularly surprising. Good aggregated subjective wellbeing may mask large sub-territorial inequalities and ignore who is missing in national or regional figures, including those intentionally keeping off-radar but who most likely experience marked disadvantage and low wellbeing. Given the population below radar will vary by place, comparisons may be misleading, although the extent people are missing and invisible usually reflects other local inequalities that could be included. Inequality and aspects of community wellbeing are likely to intersect significantly given the known social gradient in participation in civic life (Li et al., 2005). Moreover, inequalities in material and social resources may matter as much as, if not more than, absolute levels in their impact on outcomes such as health and subjective wellbeing (Wang et al., 2019; Wilkinson and Pickett, 2010). In the United Kingdom, higher inequality in subjective wellbeing within local authorities was associated with a higher vote to leave in the EU referendum (Abdallah et al., 2017) complementing arguments that increased social and geographical inequalities following austerity policies drive a growing populist nationalism (Dorling and Tomlinson, 2019). The effects of relative status appear to operate, at least in part, through social comparisons enacted over a range of everyday spaces, not just residential neighbourhoods (Wang et al., 2019).

The neglect of the temporalities of subjective wellbeing is also surprising given the focus on monitoring performance and progress over time and the global attention directed towards the Sustainable Development Goals. The current renewed interest in wellbeing in the UK initially positioned wellbeing as inseparable from the twin goals of a healthy future economy and a healthy future environment (DEFRA, 2005; NEF, 2005). This focus, however, has disappeared in all but a few frameworks (see The Happy City Index; OECD, 2015). At the same time, the growing profile in popular media of an impending global environmental crisis is associated with an upturn in environmental related anxieties, variously termed eco-anxiety or climate anxiety (Beddington, 2019; Fawbert, 2019). The mental health of young people in particular may suffer negative impacts from information circulating on the realities of the climate crisis and the implications for their futures (Taylor and Murray, 2020). Psychologists, exploring how attitudes to climate debates influence ecological action and mental health, propose strategies for anxiety management (Doherty and Clayton, 2011; Helm et al., 2018; Moser, 2020) while geographers, exploring how people manage their health and wellbeing in various conditions of adversity, propose a concept of ‘hopeful adaptation’ (Power et al., 2019).

Others, however, argue anxiety is an appropriate response, including environmental activist Greta Thunberg (at Davos, 2016):

“Adults keep saying: “We owe it to the young people to give them hope.” But I don't want your hope. I don't want you to be hopeful. I want you to panic. I want you to feel the fear I feel every day. And then I want you to act.”

This highlights a third area of neglect, that of the intersection of positive and negative experiences in constituting subjective wellbeing. The literature on subjective wellbeing is, effectively, one-sided and unbalanced. First, it treats positive and negative wellbeing as two distinct states. Secondly, as Thunberg indicates, negative emotions are not necessarily attitudinal states needing correction as undesirable and debilitating experiences. Significantly, at least in English, we lack a vocabulary for wellbeing's opposite. The preference of some authors for ‘wellness’ (Dale and Burrell, 2014; Prilleltensky, 2011) allows the opposite of unwellness, in addition to reasserting the presence of the body in what can be rather disembodied discussions of wellbeing. Dale and Burrell (2014) argue that not only must we attend to both wellness and unwellness, but to both as ‘organised embodiment’ in order fully to analyse how the ways we live, work and play come literally to occupy us differentially across populations, settings and the life-course.

The neglect of balance or trade-offs in wellbeing over time extends to intergenerational subjective wellbeing, which has had almost no direct attention, although New Zealand's emerging policy may do so (New Zealand Treasury, 2019). Existing work on intergenerational transfers in reproducing structures of inequality mainly looks at concrete resources and neglects the transfer of meanings, values and relations (Bird, 2007). Social issues in the UK, related to employment, higher education fees and debt, pensions, the EU referendum and environmental concerns, reveal major generational tensions with likely differentiated impacts on collective wellbeing. A study of social and cultural determinants of wellbeing in poor neighbourhoods in Malta demonstrates how generations had marked differentiated experiences of how social processes impact on their health and wellbeing (Satariano, 2019).

The interactions between different forms of wellbeing are also under-researched. The balance of quick wins and longer-term goals, of pleasure or hedonic wellbeing with purposeful or eudaimonic wellbeing, may be critical for long-term sustainability (Carlisle et al., 2012). Psychologists describe a consistent and robust preference in human subjects for smaller, immediate rewards over larger but deferred rewards. Modern consumerism exploits this but with likely long-term costs for individuals, communities and the planet. This also challenges government in managing local conflicts and interests when allocating both resources and benefits. The imperative to optimise subjective wellbeing through constant striving for improvement also contributes to this tension. It may, however, be more important for governments and individuals to maintain and protect existing levels of wellbeing, particularly where conditions are deteriorating following historical deindustrialisation or other economic decline, environmental degradation, green belt housing schemes or population relocation schemes. Wellbeing research rarely discusses the idea of satisficing, despite using concepts such as contentment or satisfaction. Reworking wellbeing to mean a sense of satisfaction from sustainability in lifestyle, from being comfortable beyond sufficiency but not excessively so may be crucial in redressing some of its toxicity as a concept.

### 2.4 Wholesome tonics?

This interrogation of the dominant ways of assessing and measuring individual and collective subjective wellbeing argues that how we do this may undermine how we feel about our selves, our lives, our communities and our futures. How we do this also neglects dimensions to our subjective wellbeing that should be of primary concern. The paper has referenced the indicators for subjective wellbeing in the United Kingdom since these benefited from a considerable investment of research, thinking and testing, including a nationwide consultation on what matters to people (Allin and Hand, 2017). The ONS national subjective wellbeing indicators have performed poorly in detecting negative impacts on the collective subjective wellbeing of the nation that other sources of evidence describe in response to a period of marked national political turmoil, exposure of major divisions in the country and on-going narratives of crises across social and environmental domains.

The direct toxic effects of how we understand and assess wellbeing relate to how these make us less, not more, satisfied with our lives. This operates through at least four overlapping processes: increased pursuit; constant optimisation; comparison and competition; self-blame for shortfalls. Indirect toxic effects arise from how the dominant approach neglects major issues that inextricably interconnect with our wellbeing: social affiliation and relationality; structural and material inequality; temporalities including generational and sustainable relations; balances between different types of wellbeing. These toxicities emerge from a framing of subjective wellbeing through a hyper-individualised and thwarted self, a supermarket model of social resources and an overemphasis on individual or local level action embedded in a wider context of liquid modernity and soft capitalism. Table 2 summarises these direct and indirect processes characterising our current and toxic understanding of subjective wellbeing.

**Table 2:** Toxic subjective wellbeing.

Action	Characteristics	Processes
*Direct*	Hyper-individualised and thwarted self Supermarket of social resources	Increased pursuit and focus on our own wellbeing
		Constant and unending optimisation of our own wellbeing
		Comparison and competition with others
		Self-responsibility and self-blame for short-comings
*Indirect*	Hidden, siloed and neglected factors	Relationality seriously underplayed
		Structural and material inequalities treated separately
		Temporalities of inter-generationality and sustainability omitted

The social sciences offer alternative conceptions of the self as relational, affective, caring, interdependent and inter-debted (see Gergen, 2009; McCormack and Salmenniemi, 2016; White, 2017). This inverts the logic of the supermarket model and recognises what most people already know intuitively; that the wellbeing of another entity is equally significant, sometimes more so, than our own. Support for this contention comes from research in different cultural contexts that describes how people most often endeavour to act ethically for the wellbeing of their social affiliations rather than solely in relation to their own wellbeing (White, 2018). A relational, social and moral version of wellbeing, however, is not something easily captured, particularly through a dashboard of individually directed indicators. Geographical contributions bring a disciplinary focus on the co-constituting interactions of space, place, time and wellbeing, through engagements with relationality (Bell et al., 2015), assemblage (Conradson, 2005; Duff, 2014), scale (Cummins et al., 2007; Schwanen and Wang, 2014), inequalities (Ballas and Dorling, 2013; Curtis et al., 2017) and time in terms of the life-course (Pearce, 2018), intergenerational relations (Satariano, 2019) and the wider relations of a sustainable environment (Kangmennaang and Elliott, 2018). This work, in different ways, redirects and revalorises our attention onto the social as central in productively advancing a more ‘wholesome’ understanding of subjective wellbeing.

The capture of the interrelationships of different spatial and temporal scales of life with subjective wellbeing is most familiar through the analysis of patterns and associations afforded by large data sets. Geographers have a long history of exploring spatial and social inequalities, although specific attention to inequalities in wellbeing and the association of these with other outcomes is relatively recent (Ballas and Dorling, 2013). Geographers are also among those calling for inequality to become an indicator of wellbeing (Atkinson et al., 2019; Kangmennaang and Elliott, 2018) rather than the mediator, or even mitigator, of other inequalities (see Ryff, 2017). Geographic research on temporalities in wellbeing beyond trends remains limited, but demands greater engagement in the current climate of intergenerational tension and inequality. The dominant engagement with young people's poor mental health through individually targeted therapies and resilience building (DoHE, 2018) illustrates well the neglect of subjective wellbeing a social phenomenon of spatial, scalar and temporal relations, including major contemporary issues of poverty, widening inequalities, gender identities and relations and environmental crisis.

The intimacies of relational encounters for capturing collective subjective wellbeing are challenging. Philosophical explorations of how we hope collectively attempt to bridge the hyper-individualism of positive psychology with institutional processes and mirror this paper in documenting toxic forms as well as productive forms of collective hope (Braithwaite, 2004; Drahos, 2004). More radical approaches draw on post-human, non-representational and assemblage thinking that shift the focus in wellbeing research away from a centred, individual subject and onto a complex mesh of relations, affects and trajectories (see Andrews, 2018; Bell et al., 2018; Foley, 2018). The routine of everyday sea swimming, for example, entangles connections and interactions of place, history, class, bodies, movement and habit into an account of the sedimented accretion of wellbeing over time (Foley, 2018). Wellbeing, in such approaches, emerges as an effect of the relations within which the individual is entangled (Atkinson, 2013), an ‘intra-active’ wellbeing (Smith and Reid, 2017), with very different implications for the location of wellbeing, of responsibility for wellbeing and of intervention targets. Such relational geographies of subjective wellbeing both counter and explain the toxicity of an engagement with subjective wellbeing underpinned by a hyperindividualised and thwarted self and a supermarket model of the social.

## 3 Conclusions

The paper identifies a double assumption at the heart of how we understanding subjective wellbeing: a hyper-individualised but also thwarted self; as primarily self-interested such that social affiliations serve as resources. These generate further assumptions of the desirability of positive emotions over negative ones, the apparently unassailable value of hope in the future and the responsibility of the self for improving the self. These assumptions reflect and support the role of wellbeing within the new managerial practices of soft capitalism, and, as such, are deeply entrenched in the contemporary culture of the political economy. The argument is that practices of individual and collective subjective wellbeing premised on these assumptions result in a number of toxic outcomes and an impoverished understanding of what it is to be human. In doing so, it allows the neglect of complex, enduring and iniquitous processes through which lives, individually and collectively, become differentiated. Moreover, subjective wellbeing may not just reflect and feed wider contemporary ideologies, but actively function as a significant distraction from the very real, material and increasing inequalities structuring our societies.

Where do such reflections leave us in returning to the opening provocation of whether the concept is now too toxic to be valuable or whether it remains amenable to recovery and redirection through the tonic of social science's more wholesome relational insights? The claim on subjective wellbeing as a specified set of practices and achievements commensurate with contemporary ideals of citizenship is surely too deeply entrenched in our popular imaginings and organisational practices to mount a counter-conceptualisation. The richer possibilities on offer through relationality, assemblage, post-human and non-representational theories have had little traction with wellbeing policy and practice. The embedding of the dominant understanding of subjective wellbeing within a wider political economy of liquid modernity and soft capitalism indicates this is likely to continue the case. The option of dispensing with the concept altogether to find another, less toxic, route into discussing the enrichment of our subjective and intra-active beings and becomings does, indeed, appear the more promising and productive pathway intellectually. Nonetheless, regardless of our decision, the use of the entrenched current understanding of subjective wellbeing will endure and continue to produce its negative consequences. Paradoxically, while the toxic practices of wellbeing provoke jettisoning its use, simultaneously they ethically oblige our continued prosecution of critical wellbeing research, through relational and ‘wholesome’ engagements that centre the social and keep alive, however faintly, the possibility to support more interdependent lives.

## Credit author statement

This is a single authored paper. The work is 100% by the single author.
